# The sensitivity of hospital coding to prices: evidence from Indonesia

**DOI:** 10.1007/s10754-021-09312-7

**Published:** 2021-09-07

**Authors:** Martin Chalkley, Budi Hidayat, Royasia Viki Ramadani, María José Aragón

**Affiliations:** 1 Centre for Health Economics, University of York, York, UK; 2 Center for Health Economics and Policy Studies (CHEPS), Faculty of Public Health, Universitas Indonesia, Kota Depok, Indonesia

**Keywords:** Prospective payment system, Diagnosis-related groups, Clinical coding, Indonesia, Insurance, Health, C1 (Econometric and Statistical Methods and Methodology: General), I13 (Health Insurance, Public and Private), 18 (Government Policy · Regulation · Public Health)

## Abstract

This study examines a newly introduced DRG system in Indonesia. We use secondary data for 2015 and 2017 from Jaminan Kesehatan Nasional (JKN), a patient level dataset for Indonesia created in 2014 to record public and private hospitals’ claims to the national health insurance system to investigate whether there is an association between changes in tariffs paid and the severity of inpatient activity recorded in hospitals. We find a consistent small, positive and statistically significant correlation between changes in tariffs and changes in concentration of activity, indicating discretionary but limited coding behaviour by hospitals. The results indicate that reducing price differentials may mitigate discretionary coding, but that the benefits of this are limited and need to be compared to the potential risk of having to rebase all prices upwards.

## Introduction

Diagnosis Related Groups (DRGs) (Office of Technology Assessment, 1983) based payment systems reimburse hospitals for the services they provide according to a previously defined fee for each DRG (Langenbrunner et al., 2010). They are advocated as a means of ensuring accountability and measurement of hospital activity and have been shown across a number of jurisdictions to improve efficiency and increase activity without negatively impacting on quality (Busse et al., 2011). They are, however, also associated with unintended consequences (Cots et al., 2011), including the intentional manipulation of the coding of patients by hospitals to increase their income (Steinbusch et al., 2007). This process of changing coding has been discussed by a number of different terms; gaming, upcoding and DRG ‘creep’ being the most common. In all cases the concern is that patients are assigned to a DRG with a higher tariff than the one to which they would be assigned based on their diagnoses (Kirch, 2008) with this being facilitated by the fact that DRG systems often use the severity of a patient’s condition, as determined by the provider, as a part of classification for payment. This phenomena has been discussed since the 1980s (Simborg, 1981), including analyses of its importance in driving hospital payments in the US (Carter et al., 1990; Steinwald & Dummit, 1989) and the potential solutions (Kuhn & Siciliani, 2008, 2013). A key driver of decisions within DRG-based payment are the relative prices of the different DRGs and this also applies to discretionary coding choices. Where a more severe condition has a high price relative to its low severity counterpart, there is a stronger incentive for hospitals to classify patients as high severity cases.

The setting for this study is Indonesia’s DRG system. In 2014 Indonesia introduced a national health insurance system, called *Jaminan Kesehatan Nasional* (JKN) managed by the Social Security Administering Body of Health (*BPJS Kesehatan*). The JKN is the unification of four existing types of health insurance: (1) *Jamkesmas* – the government-financed health insurance programme targeting the poor and near-poor segment of the population, (2) *Askes* – the health insurance scheme for civil servants and pensioners, (3) *Jamsostek* – the insurance scheme for formal sector workers, and (4) *Jamkesda* – the local health insurance funded by local government budget. JKN initially covered 47% of the Indonesian population in 2014, increasing to 53% in 2015, 61% in 2016, 67% in 2017 and to 75% in 2018 (Agustina et al., 2019).

Before JKN was introduced (in 2012), the utilisation of inpatient services was low with only 1.9% of the Indonesian population accessing them and bed-occupancy rates were around 60% in 2015 (Mahendradhata et al., 2017). Inpatient utilization of the Indonesian population under JKN increased to 3.1% in 2017 and 3.5% in 2018 (DJSN dan BPJS Kesehatan, 2020).

At the same time as JKN was introduced, a DRG-based payment system for hospitals was established covering both outpatient and inpatient services. This system, called Indonesian Case Base Groups (INA-CBG), had 789 categories for inpatients group and 288 categories for outpatient groups (Minister of Health, 2014b). The 789 categories for inpatients represent 263 DRGs with three severity levels each based on comorbidities and complications (I = mild—no comorbidities/complications, II = moderate—mild complications/comorbidities, III = high—major comorbidities/complications). The allocation of patients to DRGs under INA-CBGS followed the United Nations University (UNU) casemix grouper. Ministry of Health has started to collaborate with professional associations to develop the Indonesian grouper (INA-Grouper) which reflects patterns of clinical practice and resource use in Indonesia since 2016. In the future the INA-grouper will be used to replace the UNU-Grouper. While Tariff per INACBGS is created from the average cost per department, cost per day is assumed to be the same for the same department. The allocation of costs from intermediate cost to final cost is based on the number of days of care and visits.

The present study aims to examine evidence of discretionary coding in the coding in JKN utilising a crucial feature of this system; that the prices within it were adjusted since their introduction. With any new system, hospitals can be expected to adapt their reporting to generate more income. Anticipating this, price-setters in Indonesia adjusted the price differentials between high and low severity conditions but did so differentially across DRGs. The hypothesis we seek to test is that this led to lower incidence of reporting of high severity cases, according to the extent of the reduction in the price differential. Confirmation that hospitals respond in this way is evidence both of discretionary coding taking place and of the ability of setting differential prices to regulate that. To test this hypothesis we analyse data exploiting the fact that the relative prices for different severities of conditions were subject to revision between 2015 and 2017.

Whilst discretionary coding has been extensively discussed and evidenced across a number of health care systems especially in the US Medicare system, existing studies rely on innovations in the coding system to consider whether increasing the number of classifications results increased reporting of severity (Cook & Averett, 2020). These studies are largely concerned with the impact of incentives on coding within specific DRGs whereas we consider an entire DRG system and exploit differential changes between DRGs. We are thus able to give a system-level view of discretionary coding. Most DRG systems where upcoding has been examined are well-established, for example across a number of European countries (Busse et al., 2011), and have price setting that is focussed on matching financial resources to costs. The system we study is still in its infancy and provides an unusual instance of price-setters anticipating upcoding and pre-emptively adjusting prices to control it. Hence our approach is novel both in respect of its whole system focus and its use of price variation within a given DRG framework. The results support our hypothesis; we consistently find a small, positive and statistically significant correlation between changes in tariffs and changes in concentration of activity, indicating discretionary but limited coding behaviour by hospitals. The results indicate that reducing price differentials may mitigate discretionary coding, but that the benefits of this are limited and need to be compared to the potential risk of having to rebase all prices upwards.

## Indonesian case base groups (INA-CBG)

Under the JKN program, primary health care services are paid using capitation and non-capitation while hospital services are paid using DRGs or namely as INA-CBG. A standard tariff for health care services within INA-CBG is regulated by the Indonesian Ministry of Health in consultation with other government bodies. There have been four versions of the DRG tariffs. The first standard tariff regulation was in 2013 (Minister of Health, 2013) and established that tariffs differed not only by services provided (DRG/severity level) but also by region, hospital type (denoted A, B, C or D), and the class of care to which the patient was entitled (in one of three categories 1, 2, and 3). The tariff was refined in 2014 (Minister of Health, 2014b). In 2016 there were two updates to the tariff (Minister of Health, 2016a, 2016b), which added hospital ownership (public or private) to the determinants of the tariff. Private hospitals have in general a 3% higher tariff than public hospital for all types of services (Minister of Health, 2016b). In summary, since the refinement in 2016, there are 786 categories (262 DRGs with three severity levels each) for inpatient services and 289 for outpatient and these all vary across five regions, four types of hospital, two modes of hospital ownership and the three patient driven classes of care.

The five regions represent the following provinces: Banten, DKI Jakarta, West Java, Central Java, Yogyakarta and East Java (Region 1), West Sumatera, Riau, South Sumatera, Lampung, Bali and West Nusa Tenggara (Region 2), Aceh, North Sumatera, Jambi, Bengkulu, Bangka Belitung, Riau Island, West Kalimantan, North Sulawesi, Central Sulawesi, Southeast Sulawesi, West Sulawesi, South Sulawesi and Gorontalo (Region 3), South Kalimantan, East Kalimantan, North Kalimantan and Central Kalimantan (Region 4) and East Nusa Tenggara, Maluku, North Maluku, Papua and West Papua (Region 5).

Hospital type is a classification based on the range of services provided by a hospital, hospitals type A provide a wide range of subspecialist services, type B provide specialist services and limited subspecialist services, type C provide limited specialist services, and type D provide basic limited specialist services (at least internal medicine, surgery, paediatric medicine, and obstetric services), general medicine and dental healthcare services (Mahendradhata et al., 2017; Minister of Health, 2014a). Teaching hospitals can be type A or type B.

The class of care on to which a patient is entitled varies according to the contributions (premiums) they make and the amenities (e.g. private room) they can access when admitted to hospital; class 1 is the group with access to better amenities and class 3 has access to the most basic amenities. These groups are subject to different tariffs, which means that in a given hospital each DRG/severity has three tariffs, one for each of the classes of care. There is no policy of patient cost-sharing applied for services provided under JKN policy. Cost-sharing will only be applied if patients request an upgrade in class of care. For instance, patients who are enrolled in class of care 2 can request to get inpatient treatment in class 1, but will have to pay the tariff difference between class 1 and class 2. These upgrades are not captured in the data.

## Methods

Since the JKN national health insurance system was introduced nationally, there is no untreated group, so we can only exploit the variation over time in the variables of interest. Not everyone was covered by the JKN when it was introduced, but we cannot use those not covered as a control group because data on claims is only collected for those covered by JKN.

We focus on the change in tariff that occurred in 2016 to investigate whether hospital activity composition changes in response to that change.

In a given year, the relationship between the activity concentration $y_{ihk}$ and the relative prices $x_{ihk}$ in DRG $i$ in hospital $h$ for patients in class of care $k$, controlling for the patients’ age and sex in DRG $i$ in hospital $h$ in class of care $k$, can be described as:

1 $$y_{ihk} = \alpha + \beta x_{ihk} + \gamma z_{ihk} + \varepsilon_{ihk}$$

We analyse changes between two periods using a regression model in differences. Using $\Delta$ to denote the difference in a variable between two time periods, we write this model as:

2 $$\Delta y_{{ihk, \left( {t1,t0} \right)}} = \beta \Delta x_{{ihk,\left( {t1,t0} \right)}} + \gamma \Delta z_{{ihk,\left( {t1,t0} \right)}} + \Delta \varepsilon_{{ihk,\left( {t1,t0} \right)}}$$

where the dependent variable $\Delta y_{{ihk, \left( {t1,t0} \right)}}$ is a measure of the change in the activity concentration (across different severities) in DRG $i$ in hospital $h$ for patients in class of care $k$ between periods $t0$ and $t1$, $\Delta x_{{ihk,\left( {t1,t0} \right)}}$ is the variation in the relative prices for DRG $i$ in hospital $h$ for patients in class of care $k$ between periods $t0$ and $t1$ and $\Delta z_{{ihk,\left( {t1,t0} \right)}}$ is the variation in the proportion of patients in 5-year age bands and sex groups DRG $i$ in hospital $h$ in class of care $k$ between periods $t0$ and $t1$.

To measure activity concentration, we consider two approaches, one based on the coding of each hospital and one based on the coding of a grouping of hospitals facing the same tariffs for a given DRG/class of care combination, i.e. hospitals in the same region, of the same type and same ownership.

The measure of activity concentration based only on a given hospital’s activity is the percentage of activity in the highest severity level (level III) in DRG $i$ in hospital $h$ for patients in class of care $k$. This does not take into account the overall distribution of activity, for example, for a DRG where no hospital has activity in severity level III (see section Data), all hospitals will have the same value for activity concentration, zero.

The measure of activity concentration based on the activity in a hospital group is the specialisation index (Daidone & D’Amico, 2009; Gaughan et al., 2012) in DRG $i$ in hospital $h$ for patients in class of care $k$, considering the severity levels $s$ (I = mild, II = moderate, III = high) within the DRG as the activity groups. The specialisation index is based on a Gini Coefficient and ranges between zero, when a hospital’s activity is distributed as in the group of hospitals facing the same tariffs, and one, when hospital activity is fully specialised in one severity level. To simplify interpretation of results, we rescale this ratio for it to range between 0 and 100. The specialisation index shows us whether a hospital’s activity is more concentrated than in the group of hospitals facing the same tariffs; concentration can happen in any severity level, for example, a hospital with all its activity in severity level I is as specialised as a hospital with all its activity in severity level III. We interpret more concentration as indicative of less dispersion in coding of severity.

To measure relative prices we use the ratio between the highest and the lowest tariff for DRG $i$ for patients in class of care $k$ among hospitals in the same group, i.e. in the same region, of the same type and same ownership; the highest (lowest) tariff will be that of the highest (lowest) severity level for which there is activity in the hospital group, irrespective of whether one specific hospital records activity in them. For all regressions finding a statistically significant coefficient on the price variable is indicative of discretionary coding.

If the distribution of activity in respect of severity in 2015 was determined solely by patients’ need and assuming that any change in need over a short period of 2 years can be captured by our control variables then finding that the variation in the distribution of activity within a DRG is correlated with the change in the tariff for that DRG indicates that hospitals are responding to the change in tariff through their coding.

In all regressions we control for patient composition in terms of age and sex. For each hospital, we calculate the proportion of patients in a set of 5-year age groups (0–5, 6–10, …, 71–75, 76–80, 81 +) and the proportion of male patients in a given DRG $i$ and class of care $k$ combination.

The population coverage of JKN increased between 2015 (53%) and 2017 (67%) (Agustina et al., 2019), which could have an impact when comparing activity levels on these years. However, our focus is on the variation in activity between the 2 years, so we do not expect this increase in coverage to affect our methodology because even if the increase in coverage led more low (high) severity patients to have access to health care, this increase in activity in the low (high) severity level should not be correlated to the tariffs faced by hospitals.

Data analysis was performed using Stata 16 (StataCorp, 2019).

## Data

Data were derived from the *Jaminan Kesehatan Nasional* (JKN) dataset, a patient level dataset created in 2014 to record public and private hospitals’ claims to the national health insurance which has previously been used for research, mostly about healthcare utilization and evaluation on the hospital payment system (Ng et al., 2019). It has not been used to study upcoding previously (Ng et al., 2019).

Even though JKN exists since 2014, we only use data for 2015 and 2017. The 2014 data was not used as the refinement of the dataset lasted until the start of 2015, i.e. data was not submitted consistently by all hospitals in 2014 (Ng et al., 2019). While, in 2016 tariffs changed twice, in October and December, so data includes claims for different tariffs. JKN changed the way it makes data available since 2018, providing only a 1% sample of claims. Data for 2015 and 2017 allow us to compare activity under two different tariff versions. The data were extracted at the end of the year 2018. Since claims are processed between 2 months and 1 year after the admission (Ng et al., 2019), it is possible for some claims to be incurred but not reported (IBNR) in late 2017. In 2018 the maximum time to submit claims was reduced to 6 months, which indicates improved quality of the submissions, as hospitals are expected to be able to submit correct data in a reduced time frame.

The patient level data is aggregated to observations by hospital, DRG, severity level and class of care. There are 259 DRGs, three severity levels, 2405 hospitals and three classes of care. If all hospitals delivered all types of activity to all classes of care, there would be more than five million (the product of 262 DRGs, 3 severity levels, 2,405 hospitals and 3 classes of care) observations. In practice the data includes 990,097 hospital/DRG/severity level/class of care combinations, of which almost one third do not have activity in 2015 and almost one quarter have no activity in 2017. Hospital/DRG/severity level/class of care combinations with no activity in 1 year are more likely to be in private hospitals and in hospitals type D and less likely to be in hospitals type B, there are no differences across regions.

Not all hospitals record activity in all severity levels within a DRG, around 7% have no activity in the middle severity level (II) and around 14% have no activity in the highest severity level (III), and 6% of them have all their activity in the lowest severity level (I). Additionally, hospitals do not record activity in all classes of care, around 7%, 5% and 3% of them have no activity in the class of care with the highest (class of care 1), middle (class of care 2) and lowest (class of care 3) tariffs, respectively, and a small proportion of hospitals have all their activity in one class of care (1% in class of care 1, 1% in class of care 2 and 4% in class of care 3).

Tariffs are reported in currency (Indonesian Rupiah, IDR) of year 2017, therefore the variation we observe is in real terms, not due to inflation between the 2 years.

To implement our methodology, we use the severity levels $s$ within each DRG as the activity groups to calculate the two measures of $y_{ihk}$, the percentage of activity in the highest severity level and the specialisation index, and the measure of $x_{ihk}$, the ratio between the highest and the lowest tariff in a hospital group. This means that the relevant number of observations is that of hospital/DRG/class of care combinations, of which there are 581,369 observations.

The analysis dataset is not balanced, i.e. not all hospital/DRG/class of care combinations have activity in both years, around 18% of them do not have activity in 2015 and 28% do not have activity in 2017, which means that the variation between the 2 years can only be calculated for 54% of the hospital/DRG/class of care combination; the estimation sample has 316,757 observations.

## Results

### Descriptive statistics

The tariff for a hospital depends on its region, type of care and ownership; there are 40 possible combinations of region, type of care and ownership in each year (5 regions × 4 types of care × 2 ownership). The tariff also depends on the class of care (kelas = 1, 2 or 3) of the patient. This means that for each severity level in a DRG there can be more than 100 different tariffs, depending on the hospital and patient.

As an example of the variation of observed in the tariff between 2015 and 2017, we show the variation in tariff for public hospitals in region 1, with type of care A and patients in class of care 1. In Table 1, we see that for this group tariffs increased, on average, for all severity levels, but that there was a range of variation over the different DRGs and severity levels.

**Table 1:** Tariff variation between 2015 and 2017. Public hospitals in region 1, type of care A. Patients in class of care 1

	Number of DRGs	Mean % increase in tariff	Min	25th Pctile	Median	75th Pctile	Max
Severity level I	215	10.77	− 56.50	− 13.00	13.10	13.10	567.40
Severity level II	194	8.81	− 56.50	− 5.75	13.10	13.10	218.16
Severity level III	163	6.74	− 72.34	− 16.92	3.98	13.10	291.38

Our main explanatory variable is the variation in the ratio between the highest and the lowest tariff of the severity levels with activity within a DRG (1 indicates highest and lowest tariffs are the same, this happens when there is activity in only one severity level). Again, to simplify presentation, we focus on public hospitals in region 1, with type of care A and patients in class of care 1. Table 2 shows that in both years the highest tariffs are, on average twice as large as the lowest, ranging from them being equal (DRGs where only one severity level has activity) to the highest tariff being eight times as large as the lowest. In terms of variation, Table 2 shows that, on average, the difference between highest and lowest tariff decreased slightly between 2015 and 2017, with some DRGs showing larger increases/decreases between the 2 years.

**Table 2:** Ratio highest/lowest tariff by DRG. Public hospitals in region 1, type of care A. Patients in class of care 1

	Number of DRGs	Mean ratio highest/lowest tariff	Min	25th Pctile	Median	75th Pctile	Max
Tariff ratio							
2015	241	2.25	1	1.53	2.00	2.67	8.81
2017	247	2.12	1	1.50	1.90	2.58	7.96
Tariff ratio variation							
2017–2015	236	− 0.13	− 4.71	− 0.45	− 0.00	0.00	5.73

**Fig. 1:**
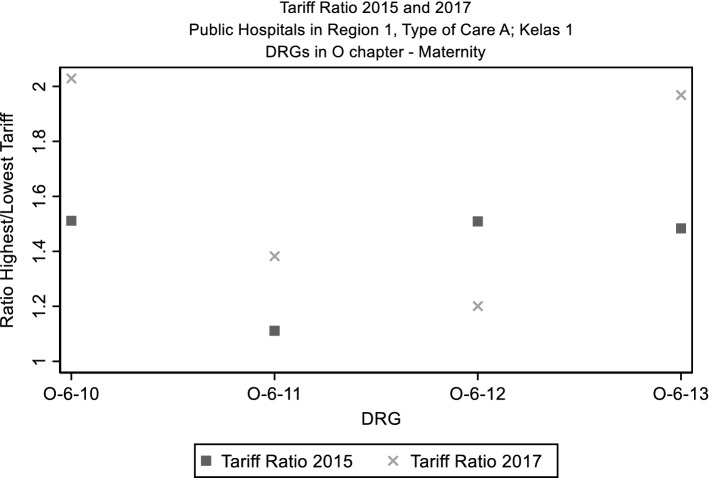
Ratio highest/lowest tariff by DRG. Public Hospitals in Region 1, Type of Care A. Patients in Class of Care 1. DRGs in O chapter (maternity)

Figure 1 shows the tariff ratio for both years for DRGs in the O chapter (maternity), where we can see that the ratio between highest and lowest tariff changes over time.

Table 3 shows the number of admissions in each year and how many of them are in the highest severity level. The decline in the percentage of patients classified as most severe from 6.05% to 5.46% can be related back to numbers of treatments in the Indonesian hospital system; it equates to 39,468 fewer patients being classified as the most severe in 2017.

**Table 3:** Number of admissions

	2015	2017
Admissions	6,157,766	6,653,823
Admissions in severity level III	372,801	363,088
% in severity level III	6.05	5.46

Table 4 shows the descriptive statistics for the estimation sample, i.e. hospital/DRG/class of care combinations with activity in both years.

**Table 4:** Descriptive statistics—estimation sample

	Mean	Std.Dev	Min	Max
Activity in severity level III—2015	6.72	16.96	0	100
Activity in severity level III—2017	5.99	15.89	0	100
Δ Activity in highest severity level^†^	− 0.73	19.26	− 100	100
Specialisation index 2015	62.24	37.53	0	100
Specialisation index 2017	61.83	38.37	0	100
Δ Specialisation index^‡^	− 0.41	44.09	− 100	100
Tariff ratio 2015^§^	290.68	159.13	100	3,172.03
Tariff ratio 2017^§^	206.85	76.76	100	1,183.39
Δ Tariff ratio^§^	− 83.82	142.37	− 3,072.01	615.67
Number of observations	316,757			

^†^Activity in Highest Severity Level is measured as the percentage of all activity in the highest severity level (III) in a given hospital/DRG/class of care combination

^‡^Specialisation Index is rescaled to range between 0 (activity distributed across severity levels as hospital group average) to 100 (all activity in one severity level)

^§^Tariff Ratios are normalised to 100, i.e. if the highest tariff is 50% higher than the lowest, this variable will take value 150

In terms of patient characteristics in the estimation sample, see Table 5, we can see that the patients admitted, overall and in severity level III, are similar in both years. The proportion of admissions of young children (age 0–5 years) and oldest patients (81 +) increased and the proportion of admissions with patients aged 76–80 years decreased between 2015 and 2017. The proportion of admissions with patients in class of care 1 (highest premiums) decreased and the proportion of admission with patients in class of care 3 (lowest premiums) increased between 2015 and 2017. The severity levels in the data are based on all comorbidities in the hospital records, but we cannot provide descriptives regarding the number of comorbidities as our data extract does not include all of them; therefore there is a risk that some of the variation is due to this unobserved characteristic, but comorbidities are implicit in the severity levels, which makes us think this risk is low.

**Table 5:** Patient characteristics – estimation sample

	All admissions		Severity level III admissions	
	2015	2017	2015	2017
Number of admissions	5,872,957	5,857,593	280,915	257,782
Age groups [% of admissions]				
0–5	10.44	13.27	9.88	10.17
6–10	3.76	4.11	4.22	4.19
11–15	3.27	3.00	3.14	2.79
16–20	5.22	4.99	2.86	2.71
21–25	7.00	7.13	2.95	2.69
26–30	8.32	7.99	3.06	2.63
31–35	8.71	8.02	3.84	3.15
36–40	7.31	6.89	4.24	3.78
41–45	6.36	5.99	5.36	5.13
46–50	7.06	6.50	7.80	7.47
51–55	7.52	7.26	9.93	10.05
56–60	7.31	7.23	11.10	11.73
61–65	6.24	6.28	10.29	11.06
66–70	4.47	4.46	7.82	8.25
71–75	3.60	3.37	6.69	6.56
76–80	3.40	0.64	6.83	1.52
81 +	0.00	2.88	0.00	6.12
Male [% of admissions]	41.78	42.16	53.35	52.86
Class of care [% of admissions]				
1	24.15	20.51	25.82	22.75
2	27.13	27.83	21.80	21.10
3	48.73	51.66	52.38	56.15

A key element in Table 4 is the decline in the ratio of prices for highest and lowest severity patients. This is reduced on average by nearly 30% (84/291) between 2015 and 2017 and therefore over the system as a whole represents a substantial reduction in the financial benefit to hospitals of classifying patients as severe. There is a corresponding reduction in the observed incidence of treatments categorised for severe patients. This is the case for both or our measures (the percentage of activity in the most severe category and the specialisation index). The percentage of activity in the most severe category in the estimation sample are slightly higher (around 0.6 percentage points) than in the full data (Table 3). The change in the specialisation index indicates that there is less variation in severity within hospital groupings in 2017 than in 2015, although the numerical magnitude of this difference is not easily interpretable. Our hypothesis is that both of these changes in severity will vary between hospitals and within groupings of hospitals according to the extent of the reduction in differential prices, where this reduction varies by DRG, hospital, and the patients’ class of care.

### Regression results

Table 6 shows the regression results of estimating Eq. (2) for all observations (left) and only for those that changed the composition of their activity (right) using as dependent variable the percentage of activity in the highest severity level. In both cases the change in the tariff, measured with the variation in the ratio between the highest and the lowest tariff in the hospital group, is statistically significant and positively correlated to changes in activity composition, measured as the variation of the percentage of activity in the highest severity. Hence the changes observed in Table 4 cannot be attributed to changing case mix or the composition of treatments undertaken by the healthcare system alone. The estimated coefficient indicates that a one point decrease in the ratio between highest and lowest tariff is associated with a decrease of around 0.003 percentage points in the activity in the highest severity level (III). Hence, after controlling for patient ages and the changing case mix of DRGs, the decline from 291 to 207 (decrease of 84 points) in the average tariff differential is associated with a reduction of 15,517 patients being classified in the most serious category. As is common in a regression in differences, the over overall fit of the model (R-squared) is low. This is indicative of a large proportion of differences in the dependent variable being attributable to characteristics of hospitals that are differenced out. Running a regression in levels, such as in Eq. (1), typically exhibits an R-squared of 0.1. More relevant in terms of the degree of explanation that Eq. (2) achieves is the fact that the price variable explains more than 39% of the total difference in the specialisation measure that is observed in Table 3.

**Table 6:** Regression results—$y$: percentage of activity in highest severity level

	All Obs	$\Delta y \ne 0$
Δ Tariff ratio	0.0030***	0.0096***
	(0.0004)	(0.0013)
Patients’ AGE AND SEx	Yes	Yes
R-squared	0.0040	0.0138
N	316,757	123,325

Standard errors, clustered by hospital, in parenthesis

***Represents 1% significance

Table 7 shows the regression results of estimating Eq. (2) for all observations (left) and only for those that changed the composition of their activity (right) using the specialisation index as dependent variable. In both cases the change in the tariff, measured with the variation in the ratio between the highest and the lowest tariff in the hospital group, is positively correlated to changes in activity composition over severity levels, measured as the specialisation index. The coefficient indicates that a one point decrease in the ratio between highest and lowest tariff is associated with a decrease of around 0.005 points in the specialisation index. Referring to Table 4, this result is consistent with the observed reduction in the specialisation index (0.41), as it estimates it to be 0.42 (84 × 0.005). The results in the second column indicate, as expected, that the responsiveness to tariff changes is focused on a subsample of hospitals and within that subsample the effect is larger.

**Table 7:** Regression results—$y$: specialisation index

	All Obs	$\Delta y \ne 0$
Δ Tariff ratio	0.0053***	0.0088***
	(0.0009)	(0.001)
Patients’ age and sex	Yes	Yes
R-squared	0.0014	0.0027
N	316,757	223,897

Standard errors, clustered by hospital, in parenthesis

***Represents 1% significance

These results indicate that decreases (increases) in relative tariffs are associated to decreased (increased) specialisation but they need to be interpreted with caution as the data is relatively noisy as there are hospital/DRG/class of care combinations with low levels of activity and many of them (46%) have activity in only 1 year.

### Sub-sample analysis

We repeated the analysis focusing on sub-samples defined by hospital ownership, region, hospital type and class of care. Table 8 summarizes the sub-sample results, showing the overall coefficients for the variable of interest, $\Delta$ Tariff Ratio, and the (significant) coefficients in the different sub-samples. The overall nature of results is preserved over different settings but the magnitude of coefficients varies. For example, the coefficient for $\Delta$ Tariff Ratio is larger in private hospitals than in public hospitals and it varies by region. For the hospital type offering most specialised services (A), there is no significant correlation between variations in the tariff and activity concentration, but there are some differences between the other hospital types. For different patient classes of care, there are less differences between them when using the proportion of activity in the highest severity level than when using the specialisation index.

**Table 8:** Regression results—subsamples

	$y$: Activity severity level III		$y$: Specialisation index	
	All Obs	$\Delta y \ne 0$	All Obs	$\Delta y \ne 0$
Overall coefficient (Tables 6 and 7)	0.0030	0.0096	0.0053	0.0088
Hospital ownership				
Public	0.0011	0.0037	0.0046	0.0076
Private	0.0060	0.0174	0.0065	0.0107
Region				
Region 1	0.0023	0.0074	0.0027	0.0044
Region 2	0.0059	0.0193	0.0094	0.0151
Region 3	0.0042	0.0148	0.0094	0.0154
Region 4	–	–	0.0496	0.0681
Region 5	0.0149	0.0487	0.0221	0.034
Type of care				
A	–	–	–	–
B	0.0035	0.0087	0.0052	0.0073
C	0.0025	0.0090	-	-
D	0.0058	0.0287	0.0243	0.0433
Class of care (Kelas)				
1	0.0024	0.0081	-	-
2	0.0035	0.0121	0.0050	0.0091
3	0.0032	0.0090	0.0095	0.0136
Providers with Activity > Median	0.0029	0.0081	0.0029	0.0043
Excluding outliers (> 95th pctile)	0.0061	0.0170	0.0116	0.0170

Table only shows estimators of the coefficient for Δ Tariff Ratio significant at the 5% level

We also restricted the sample to the largest hospitals (i.e. with activity greater than the median) in each DRG/class of care combination. The results using the percentage of activity in severity level III as dependent variable are similar to those in the whole sample. Using the specialisation index as dependent variable, we observe differences, but we do not re-calculate the specialisation index in the restricted sample.

We also repeated the analysis excluding from the sample the observations where the ratio between the tariff for the lowest and highest severity level exceeds the 95th percentile of the distribution. The results show coefficients larger (around twice as large) than those observed in Tables 6 and 7.

Taken together the regression results indicate a consistent positive and statistically significant correlation between the change in tariff ratio and both of our measures of severity of coding and thus indicate both discretionary coding behaviour by hospitals and that this varies according to the extent to which prices show dispersion across different severities.

## Discussion

The phenomena of hospitals reporting conditions as more severe – upcoding – is a much-discussed unintended consequence of the adoption of DRG systems. It implies that the financing cost of hospital services increases over time due to manipulation of the coding system by hospitals seeking higher reimbursement. Whilst most DRG systems have been observed to experience upcoding (or ‘creep’) over time it is difficult to separate the drivers of that as between increasing severity of illness and hospital behaviour. This is because in most systems, prices are calibrated to costs and innovation comes in the form of changing the number or definition of DRG to allow for increasing complexity of health conditions. Increased reported severity can therefore be a consequence of severely ill patients being correctly classified according to a new and more sensitive classification system. In contrast to this we study a system in which there has been explicit price variation and examine the response of reported severity to changing price differentials. This provides a novel and potentially informative indication of discretionary coding because if severity were purely a matter of patients’ health conditions, we would not expect reported severity to change as prices change. The numerical responsiveness of coding severity to prices differentials then gives an indication of how much influence price setters can have over discretionary coding. The present study uses an opportunity to implement this approach by examining data on a newly instigated DRG system in which price setting was proactively used to pre-empt a potential move to upcoding.

Our findings provide consistent evidence of discretionary coding in the Indonesian system. Reductions in the price differential between most and least severe patients are associated with reductions in the proportion of patients recorded in the most severe category. This is true both overall and for different types of hospital, different payment categories of patient and across different regions. In all cases the finding is of statistically significant associations in the direction expected. We further find evidence that the discrepancies between similar hospitals in terms of their coding are reduced when price differentials are reduced, which is suggestive of different hospitals being more or less prone to discretionary coding, but these differences are alleviated when the financial incentive to up-code is reduced.

Our findings in respect of magnitude of pricing effects are both novel and potentially important from the perspective of health policy in regard to DRG systems. In the Indonesian system with 6.65 million treatments in 2017, 363,088 patients were placed in the most severe category. Simply in terms of descriptive statistics this is 10,000 less than in 2015 (372,801 admissions in the highest severity level), but in that year only 6.16 million (10% fewer) cases were treated. Hence, in terms of comparing like with like, 39,468 fewer cases were classified as most severe in 2017 compared with what might have been expected based on 2015 figures. Between these 2 years the price differentials between most and least severe patients were reduced by 30%. It is important to note that a considerable proportion of the reduction is attributable to factors other than changing prices. The regression estimates imply that 15,517 (84 point reduction on tariff ratio, 0.003 estimated coefficient and 6,157,766 patients in 2015) of the 39,468 reduction is associated with the price changes. Very large (30%) reductions in differential prices are, therefore, associated with very small (0.6%) reductions in the number of patients coded as most severe. The determination of prices in DRG systems is complex and price setters face a number of trade-offs. If price differentials between high and low severity patients are too small to compensate hospitals for their differential costs then it may be necessary to increase prices on average to prevent hospitals from running deficits. Our results show that the benefit of reducing price differentials may be very small and that needs to be compared to the potential risk to the overall cost of the system of having to rebase all prices upwards.

The main limitation of this study is the lack of a treatment group and, therefore, depending only on variation over time to identify evidence of upcoding. Additionally, the unbalanced nature of the data, not all hospitals/DRG/class of care combinations have activity in both years, reduces the estimation sample.

The key policy implication of our study is in relation to setting DRG prices. We have established that price differentials for different severity DRGs are associated with changes in coding behaviour. This gives both an additional instrument and an additional constraint for policy makers in their role of price setting. When prices are viewed as a mechanism for transferring resources to finance healthcare, it is natural to align prices to the costs of treatment. This is common practice in many DRG systems. Where there are DRGs that correspond to different severities of the same condition this may imply setting a much higher price for the treatment of severe patients. Our results show that this may then increase the incidence of claims for severity. Hence, policy makers need to balance the requirements of transferring resources with the risks of facilitating discretionary coding and prices may need to differ from costs – paying more than cost for less sever patients and vice versa.

## Data Availability

We thank Badan Penyelenggara Jaminan Sosial (BPJS) Kesehatan for providing the JKN dataset. Researchers interested in using this dataset can find details in https://bpjs-kesehatan.go.id/bpjs/ and https://data.bpjs-kesehatan.go.id/bpjs-portal/action/landingPage.cbi
